# Visceral Obesity and Its Association with Severe Coronary Artery Calcification in Patients with Metabolic Dysfunction-Associated Steatotic Liver Disease

**DOI:** 10.3390/diagnostics14202305

**Published:** 2024-10-17

**Authors:** Min Kyu Kang, Jeung Eun Song, Young Oh Kweon, Won Young Tak, Soo Young Park, Yu Rim Lee, Jung Gil Park

**Affiliations:** 1 Department of Internal Medicine, College of Medicine, Yeungnam University, Daegu 42415, Republic of Korea; kmggood111@naver.com; 2 Department of Internal Medicine, School of Medicine, Daegu Catholic University, Daegu 42472, Republic of Korea; ssong3004@naver.com; 3 Department of Internal Medicine, School of Medicine, Kyungpook National University, Kyungpook National University Hospital, Daegu 41994, Republic of Korea; yokweon@knu.ac.kr (Y.O.K.); wytak@knu.ac.kr (W.Y.T.); psyoung0419@gmail.com (S.Y.P.)

**Keywords:** atherosclerotic cardiovascular disease, coronary artery calcification, metabolic dysfunction-associated steatotic liver disease, visceral obesity

## Abstract

**Background/Objectives**: The role of body composition parameters in patients with metabolic dysfunction-associated steatotic liver disease (MASLD) with presence and severity of coronary artery calcification (CAC) is still not fully elucidated. We aimed to evaluate the impact of computed tomography (CT)-based body composition parameters in patients with MASLD with CAC severity. **Methods**: In this multicenter study, 1870 individuals underwent cardiac CT for the detection of CAC as well as ultrasonography for the diagnosis of hepatic steatosis. The presence of CAC was defined by a CAC score threshold of >0, while severe CAC was defined by a threshold of >300. Using the abdominal cross-sectional CT images at the L3 vertebra level, we analyzed the skeletal muscle index, visceral to subcutaneous adipose tissue ratio, and muscle density using the Hounsfield unit. **Results**: Of 648 patients with MASLD, the proportions of presence of CAC and severe CAC were 45.2% and 9.9%, respectively. Visceral obesity was not associated with the presence of CAC after adjustment for age, sex, smoking, statin therapy, type 2 diabetes, and advanced fibrosis (adjusted odds ratio (aOR), 1.38; 95% confidence interval (CI), 0.86–2.23; *p* = 0.180). However, visceral obesity was independently associated with severe CAC after adjustment for several metabolic risk factors (aOR, 3.54; 95% CI, 1.25–14.90; *p* = 0.039), and adjustment for atherosclerotic cardiovascular disease risk scores (aOR, 3.74; 95% CI, 1.31–15.79; *p* = 0.032). **Conclusions**: Visceral obesity may serve as a novel prognostic CT-based radiological biomarker for patients with MASLD with severe CAC.

## 1. Introduction

Non-alcoholic fatty liver disease (NAFLD) affects approximately 30% to 35% of the global population and is strongly associated with obesity, insulin resistance (IR), hypertension, dyslipidemia, and type 2 diabetes [1,2,3]. These associations significantly elevate the risk of cardiovascular disease (CVD) [2,4,5,6]. However, the heterogeneous pathogenesis and ambiguity in NAFLD classification often lead to an underestimation of the impact of associated metabolic comorbidities and extrahepatic manifestations [7]. To address these issues, the nomenclature has recently shifted from NAFLD to metabolic dysfunction-associated steatotic liver disease (MASLD) [8,9]. MASLD is defined by hepatic steatosis accompanied by at least one of five cardiometabolic criteria in the absence of other evident causes, underscoring its connection with metabolic diseases and CVD [8,9].

Quantitative assessment of coronary artery calcification (CAC) provides a dependable, non-invasive method for identifying coronary artery disease (CAD) and assessing the severity of CVD in asymptomatic patients. CAC scoring is strongly linked to the degree of atherosclerosis and cardiovascular mortality, regardless of conventional risk factors [10,11,12].

Computed tomography (CT)-derived body composition metrics—such as skeletal muscle, visceral adipose tissue (VAT), subcutaneous adipose tissue (SAT), and myosteatosis—are increasingly recognized as prognostic indicators for mortality and liver-related and cardiovascular morbidities in chronic liver disease patients [13,14,15]. Previous studies have demonstrated that sarcopenia is related to advanced fibrosis and subclinical atherosclerosis in MASLD patients [14,16]. Furthermore, VAT has been linked to histologic severity and advanced fibrosis in biopsy-proven MASLD [13]. Visceral obesity and sarcopenia share a pathophysiologic mechanism involving IR and chronic inflammation, contributing to MASLD progression and increased CVD risk [4,17,18]. Despite these findings, research examining whether visceral obesity or sarcopenia more significantly impacts cardiovascular outcomes in patients with MASLD remains limited. Additionally, the clinical significance of these CT-based body composition parameters in relation to CAC presence and severity in patients with MASLD has not been thoroughly investigated.

In the current study, we assessed sarcopenia, visceral adiposity (utilizing the visceral-to-subcutaneous adipose ratio [VSR]), and myosteatosis (quantified using Hounsfield units [HU]) to examine their impact on the severity of CAC in patients with MASLD.

## 2. Materials and Methods

### 2.1. Study Participants

**Figure 1:**
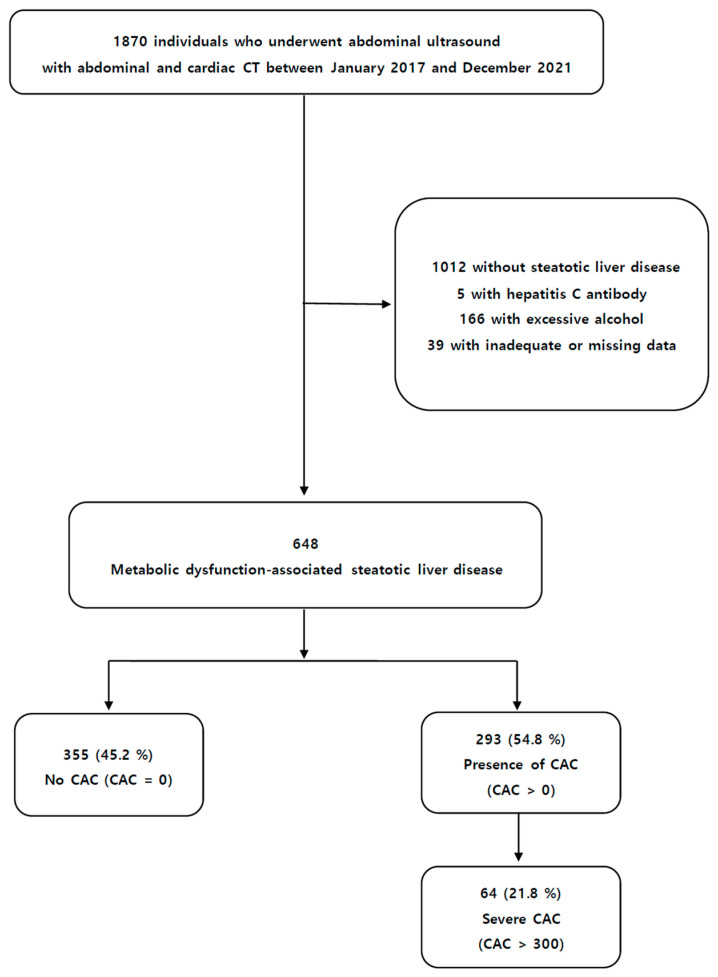
Flowchart of the enrolled patients.

This cross-sectional, retrospective, multicenter study included participants who underwent a comprehensive evaluation, which incorporated ultrasound and both abdominal and cardiac CT, between January 2017 and December 2021 at two health-promotion centers. A total of 1870 individuals were reclassified based on the following exclusion criteria: (i) participants without steatotic liver disease (*n* = 1012); (ii) participants who had a positive result of viral hepatitis (*n* = 5); (iii) excessive alcohol consumption (male > 210 g/week; female > 140 g/week) (*n* = 166); and inadequate or missing data (*n* = 39). A total of 648 patients diagnosed with MASLD were incorporated into the present study (Figure 1). Informed consent from participants was exempt due to the retrospective design of this study. The study protocol received approval from the Institutional Review Board at Yeungnam University Hospital (IRB No. 2022-06-007).

### 2.2. Data Collection and Definition of MASLD

All anthropometric profiles were assessed and documented by the trained staff. Information regarding alcohol consumption, medication use, and comorbidities was gathered through a self-administered questionnaire. Blood samples and abdominal imaging were conducted following an overnight fast for each participant. The patients’ biochemical and metabolic profiles were subsequently assessed.

Hepatic steatosis was evaluated using abdominal ultrasound according to the following criteria: increased hepatic echogenicity compared to the outer layer of the kidney, attenuation of the ultrasound in deeper liver regions, and obscuration of intrahepatic vessels [19]. MASLD is defined as hepatic steatosis accompanied by at least one of the five cardiometabolic criteria: (i) body mass index ≥ 23 kg/m^2^ or waist circumference > 94 cm for male and 80 cm for female; (ii) fasting glucose ≥ 100 mg/dL or HbA1c ≥ 5.7% or type 2 diabetes or treatment of type 2 diabetes; (iii) blood pressure ≥ 130/85 mmHg or antihypertensive drug treatment; (iv) plasma triglyceride ≥ 150 mg/dL or lipid lowering treatment; (v) plasma high-density lipoprotein ≤ 40 mg/dL for male and ≤ or 50 mg/dL for female lipid lowering treatment [8,9].

### 2.3. Assessment of Body Composition Parameters

Utilizing the Picture Archiving and Communications System (Centricity, GE Healthcare, Chicago, IL, USA), four body composition metrics were assessed using abdominal CT images at the third lumbar vertebra (L3). Unenhanced abdominal cross-sectional CT images for body composition were analyzed using a well-established software application (Automated Muscle and Adipose Tissue Composition Analysis) (AutoMATiCA, https://gitlab.com/Michael_Paris/AutoMATiCA; assessed 4 May 2023) [20].

**Figure 2:**
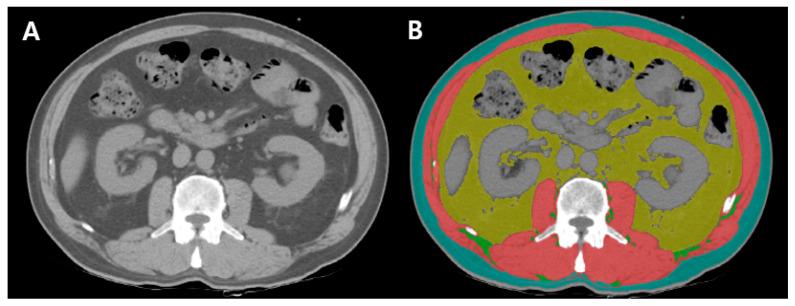
Cross-sectional CT images at the level of L3. (**A**) Unenhanced image using Picture Archiving and Communications System (**B**) body composition parameters classified by color-coded distribution areas using AutoMATiCA (Red, skeletal muscle area; yellow, VAT area; blue, SAT area; green, IMAT area [20].

Skeletal muscle area was quantified as the total area of intra-abdominal muscles, utilizing HU thresholds of −29 to 150. VAT area was measured as the fatty tissue within the intra-abdominal skeletal muscles, excluding regions occupied by the kidneys, liver, intestines, and other organs, using HU thresholds of −50 to 150. SAT area was quantified as the fat layer between the skeletal muscle and the abdominal skin, using HU thresholds of −190 to −30. Intermuscular adipose tissue (IMAT) was calculated as the fat accumulation within the intra-abdominal skeletal muscle, using HU thresholds of −190 to −30 (Figure 2) [20].

Sarcopenia was defined as a skeletal muscle index value less than 50 cm^2^/m^2^ for males and less than 39 cm^2^/m^2^ for females, calculated by dividing skeletal muscle area by the square of height (m^2^) [21]. Visceral obesity was defined as a VSR of ≥1.0 for males and ≥0.5 for females, indicating a higher proportion of abdominal adipose tissue [22]. Myosteatosis was defined as mean HUs of IMAT < 41 HUs for patients with a BMI < 25 kg/m^2^ and <33 HUs for those with a ≥25 kg/m^2^ [23]. 

### 2.4. Quantification of CAC

Non-contrast volumetric cardiac CT was performed at each institution using a 256-slice CT scanner (Revolution; GE Healthcare; Waukesha, WI, US) and a 320-slice CT scanner (SOMATOM Force; Siemens Healthineers; Erlangen, Germany). Scanning was performed at end-inspiration with subjects instructed to hold their breath. The tube voltage varied between 100 and 120 kVp, and five filter rotations were utilized for image reconstruction, resulting in 2.5 mm thick slices. CAC scores were computed for each cardiac CT protocol using the Agatston method with independent post-processing software A CAC score of 0 was defined as the threshold for defining the presence of CAC, and a score exceeding 300 was classified as severe CAC [11,24].

### 2.5. Statistical Evaluation

Continuous variables were evaluated using either Student’s *t*-test or the Mann–Whitney U test, while categorical data were compared with the chi-square test or Fisher’s exact test, following a normality assessment. To examine the relationships between body composition parameters and the presence or severity of CAC in patients with MASLD, adjusted logistic regression models employing stepwise backward elimination of odds ratios (OR) were utilized. Model 1 was adjusted for age and sex, while model 2 included adjustments for smoking, statin therapy, type 2 diabetes, and advanced fibrosis, which was defined as a fibrosis-4 index ≥ 2.67 following the adjustments made in model 1. To prevent multicollinearity, model 3 was adjusted solely for the atherosclerotic cardiovascular disease (ASCVD) risk score, which encompasses age, sex, race, smoking, blood pressure, antihypertensive medication usage, diabetes status, and levels of total and high-density lipoprotein cholesterol. Statistical evaluations were conducted using R (version 4.1.0; R Core Team, 2021; R Foundation for Statistical Computing, Vienna, Austria), with statistical significance defined as *p* < 0.05.

## 3. Results

### 3.1. Baseline Characteristics

Table 1 presents the baseline characteristics of MASLD patients with CAC and those without CAC. Of the 648 MASLD patients, 293 (45.2%) were identified as having CAC. In comparison to MASLD patients without CAC, those with CAC were older (60.0 [55.0–66.0] vs. 55.0 [49.0–60.0] years, *p* < 0.001), had a higher proportion of males (84.3% vs. 74.1%, *p* = 0.002), more likely to have type 2 diabetes (39.2% vs. 22.8%, *p* < 0.001), more likely to be current smokers (73.0% vs. 64.5%, *p* = 0.025), had high proportions of advanced fibrosis (9.2% vs. 2.8%, *p* = 0.001), had higher levels of HbA1c (5.9 [5.5–6.6] vs. 5.7 [5.4–6.1], *p* < 0.001), exhibited a greater prevalence of statin therapy (18.8% vs. 15.8%, *p* = 0.002), had a high proportion of visceral obesity (63.1% vs. 44.8%, *p* < 0.001), and showed an increased ASCVD risk scores (14.3 [9.3–22.8] vs. 8.8 [4.0–14.9], *p* < 0.001). The median CAC score was 82.5, with the prevalence of mild, moderate, and severe CAC groups reported as 56.3%, 21.8%, and 21.8%, respectively.

**Figure 3:**
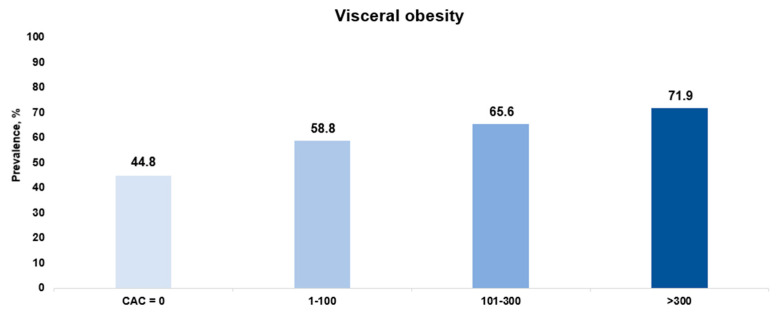
Prevalence of visceral obesity according to the severity of CAC in patients with MASLD. CAC, coronary artery calcification; MASLD, metabolic dysfunction-associated steatotic liver disease.

When classified based on the severity of CAC, 64 patients (9.9%) demonstrated severe CAC, and the median CAC score is 687.8. Compared to the individuals without severe CAC, those with severe CAC were of greater age (64.0 [61.0–69.5] vs. 57.0 [51.0–62.0] years, *p* < 0.001), more likely to have type 2 diabetes (45.3% vs. 28.6%, *p* = 0.009), had high proportions of advanced fibrosis (17.2% vs. 4.5%, *p* < 0.001), had higher levels of HbA1c (6.0 [5.5–6.7] vs. 5.7 [5.4–6.3], *p* = 0.021), exhibited a greater prevalence of statin therapy (21.9% vs. 16.6%, *p* = 0.023), had a high proportion of visceral obesity (71.9% vs. 51.0%, *p* = 0.002), and showed an increased ASCVD risk scores (19.4 [12.2–33.0] vs. 10.8 [4.0–16.9], *p* < 0.001) (supplementary Table S1). In addition, a statistically significant increase in the prevalence of visceral obesity was observed, rising from 44.8% in the no-CAC group to 58.8% in the mild CAC group, 65.6% in the moderate CAC group, and 71.9% in the severe CAC group (Figure 3).

### 3.2. Adjusted Associations of Visceral Obesity on the Presence of CAC in Patients with MASLD

In the univariate analysis, as demonstrated in Table 2, age (odds ratio (OR), 1.10; 95% confidence interval (CI), 1.07–1.14; *p* < 0.001 for severe CAC), type 2 diabetes (OR, 2.07; 95% CI, 1.22–3.49; *p* = 0.007 for severe CAC), and advanced fibrosis (OR, 4.45; 95% CI, 2.01–9.32; *p* < 0.001 for severe CAC) were consistently linked to both the presence of CAC and severe CAC in MASLD patients. Of the three body composition measures considered, only visceral obesity (OR, 2.25; 95% CI, 1.48–3.47; *p* < 0.001 for presence of CAC and OR, 5.15; 95% CI, 1.87–21.34; *p* = 0.006 for severe CAC) was associated with the presence and severity of CAC in patients with MASLD. 

We evaluated the clinical impact of visceral obesity on the presence and severity of CAC in patients with MASLD. Table 3 presents the adjusted risk of visceral obesity in relation to the presence and severity of MASLD. After adjusting for age and sex, visceral obesity was not found to be associated with the presence of CAC (model 1: adjusted OR (aOR), 1.44; 95% CI, 0.90–2.31; *p* = 0.130) and further stepwise adjustment for smoking, statin therapy, type 2 diabetes, and advanced fibrosis (model 2: aOR, 1.38; 95% CI, 0.86–2.23; *p* = 0.180). However, after adjustment for ASCVD risk scores, visceral obesity had a significant association with the presence of CAC (model 3: aOR, 1.67; 95% CI, 1.08–2.64; *p* = 0.024).

### 3.3. Adjusted Associations of Visceral Obesity on the Severe CAC in Patients with MAS

**Figure 4:**
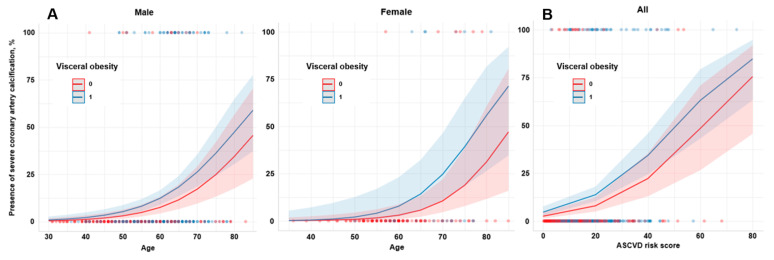
Predicted probabilities of the severe CAC (**A**) age- and sex-adjusted model, (**B**) ASCVD risk score-adjusted model. ASCVD, atherosclerotic cardiovascular disease. CAC, coronary artery calcification.

Contrary to the previous results, visceral obesity was found to be independently associated with severe CAC after adjustment for age and sex (model 1: aOR, 3.75; 95% CI, 1.32–15.77; *p* = 0.030; Figure 4A), further stepwise adjustment for smoking, statin therapy, type 2 diabetes, and advanced fibrosis (model 2: aOR, 3.54; 95% CI, 1.25–14.90; *p* = 0.039), and adjustment for ASCVD risk scores (model 3: aOR, 3.74; 95% CI, 1.31–15.79; *p* = 0.032, Figure 4B) (Table 3).

## 4. Discussion

The results of our study show that the prevalence of visceral obesity increases according to the severity of CAC in patients with MASLD. In addition, we identified that visceral obesity is associated with the presence of CAC in adjustment for ASCVD and significantly associated with severe CAC, independently of traditional metabolic risk factors. Visceral obesity may serve as a novel prognostic CT-based radiological biomarker for patients with MASLD with severe CAC. 

This study has some clinical implications. First, visceral obesity showed a stronger correlation with CAC severity than sarcopenia and myosteatosis in patients with MASLD. Several studies demonstrated that visceral obesity affects the presence or severity of CAC in general population [17,25,26]. Lee et al. demonstrated that predominance of VAT/SAT at ≥30% revealed the high prediction for CAC progression (adjusted hazard ratio, 2.20; 95% CI, 1.74–2.78), regardless of body mass index or waist circumference in a healthy Korean population [25]. In a study of 5969 patients without a history of CVD or cardiac symptoms, osteosarcopenia was associated with moderate-to-extensive CAC (OR, 2.709; 95% CI, 1.128–6.505), whereas sarcopenia was not associated (OR, 0.882; 95% CI, 0.536–1.454) [26]. However, because muscle or adipose tissue parameters were not included in the two studies, it is difficult to determine whether visceral obesity or sarcopenia has more direct clinical significance for CAC severity. In one study of patients with MASLD, hepatic fibrosis (≥ stage 1) is significantly associated with visceral fat area, not muscle mass [27]. The clinical significance of visceral fat area for MASLD progression was elucidated using both muscle and visceral fat, but there was no adjustment for individual body surface area simply based on fat area. 

In our study, visceral obesity was defined using VSR to correct for the protective metabolic effect of SAT. A positive energy balance leads to fat accumulation in the SAT, which initially has a relatively minor effect on IR in the early stages of MASLD [28]. However, as adipose tissue dysfunction and an intolerance to extensive energy develop, excess fat begins to accumulate as a form of VATS, leading to ectopic fat deposition, including the liver, heart, and skeletal muscle [29,30,31,32]. Given that blood from VAT drains into the liver through the portal vein, elevated concentrations of free fatty acids and cytokines secreted by VAT adipocytes may contribute to the development of MASLD and IR [33]. Furthermore, the interaction between the liver, adipose tissue, and pro-inflammatory molecules—such as interleukin-6 and tumor necrosis factor-alpha—released from activated macrophages and adipokines plays a crucial role in the progression of MASLD [34]. Considering these points, it can be expected that the accumulation of visceral obesity is preceded by the deposition of ectopic fat in the muscles (myosteatosis) and muscle degradation (sarcopenia) due to persistent chronic inflammation. Persistence of metabolic dysregulation due to visceral obesity may ultimately be associated with atherosclerosis.

Second, the role of visceral obesity was prominent in severe CAC in our study. CAC serves as a marker of overall coronary atherosclerotic burden and is a crucial tool in cardiovascular risk stratification and preventive treatment for asymptomatic patients with indeterminate cardiovascular disease risk [11,35]. CAC is quantified using the Agatston score, which is the sum of the attenuation using HU and the area of all CAC lesions in the coronary arteries [11]. The Agatston score is then categorized into risk levels: very low risk (CAC = 0), mildly increased risk (CAC = 1–99), moderately increased risk (CAC = 100–299), and moderate to severely increased risk (CAC ≥ 300) [36]. 

Previously, the presence of CAC (CAC = 0) was used as a criterion for increased CVD risk, but CAC = 0 has been known as a criterion with high negative predictive value for excluding obstructive CAD and high-risk CAD [35]. Recently, patients with CAC > 300 and no ASCVD events were shown to have a similar ASCVD risk as patients with ASCVD events [37]. Healthy individuals with a CAC of 300 or higher should be treated in the same manner as those with prior ASCVD, considering that the risk of major adverse cardiovascular events is equivalent to that of patients with prior ASCVD events. In addition, the evaluation of CAC scores (CAC > 300) in normal individuals may serve as a reference point for the prevention of potential future ASCVD events [37,38,39,40,41]. Considering that the presence of visceral obesity was associated with an approximately fourfold risk of severe CAC in our study, our results suggest that aggressive management of visceral obesity may be helpful in controlling future CVD risk. 

Third, we also evaluated the CAC risk associated with visceral obesity, adjusting for the presence of advanced fibrosis, defined as a Fib-4 score ≥ 2.67. It is recognized that MASLD is linked to both fatal and nonfatal CVD events, contingent upon the stage of liver fibrosis. [42]. We have previously shown that VAT is associated with advanced fibrosis in patients with Biopsy-proven MAFLD [13]. In addition, our previous research has demonstrated that significant fibrosis, as assessed using magnetic resonance elastography, is correlated with the presence of CAC in patients with MASLD [43]. The finding that visceral obesity is associated with severe CAC independently of advanced fibrosis, in addition to conventional risk factors, has important clinical implications.

Cautious interpretation is warranted due to various limitations present in our study. First, this study was a cross-sectional retrospective analysis, which does not reveal a causal relationship with CVD and visceral obesity in patients with MASLD. Second, our study included Korean individuals who voluntarily attended the health-promotion center and were able to afford the comprehensive assessment, such as abdominal and cardiac CT. This may have introduced a selection bias. Third, since non-calcified plaques are known to be more unstable and prone to rupture compared to calcified plaques, our study is unable to assess the burden of non-calcified plaques using non-contrast cardiac CT [44]. However, considering the cost-effectiveness and the potential adverse effects associated with contrast-enhanced cardiac CT, such as contrast-induced nephrotoxicity and allergic reactions, it is realistically limited to implement contrast CT for all participants at the health-promotion center who do not present with heart-related symptoms. Fourth, laboratory tests related to insulin resistance, such as homeostasis model assessment of insulin resistance and chronic inflammatory markers, such as interleukin-6 and tumor necrosis factor-alpha, were not analyzed due to the retrospective design of the study and the significant amount of missing data. Additional large-scale multinational prospective studies are required to validate the causal clinical significance of changes in body composition metrics on CVD risk in patients with MASLD.

## 5. Conclusions

Visceral obesity may be a potential prognostic CT-based radiological biomarker factor for severe CAC in patients with MASLD. Assessment of visceral obesity, independent of traditional metabolic risk factors, is valuable as a potential target for secondary prevention of future ASCVD. Additionally, controlling and managing visceral obesity may be a crucial treatment strategy for patients with MASLD who have CAC.

## Figures and Tables

**Table 1:** Baseline characteristics.

	CAC > 0 *n* = 293 (45.2%)	CAC = 0 *n* = 355 (54.8%)	*p*-Value
Demographic profile			
Age (years)	60.0 [55.0–66.0]	55.0 [49.0–60.0]	<0.001
Male	247 (84.3)	263 (74.1)	0.002
BMI (kg/m^2^)	23.1 [22.0–26.0]	23.0 [22.0–26.7]	0.860
Waist circumference (cm)	89.2 [85.0–95.0]	90.2 [85.7–96.0]	0.308
Type 2 diabetes	115 (39.2)	81 (22.8)	<0.001
Hypertension	199 (67.9)	222 (62.5)	0.178
Current smoker	214 (73.0)	229 (64.5)	0.025
Biochemical profile			
AST (U/L)	28.0 [22.0–37.0]	27.0 [21.0–36.0]	0.190
ALT (U/L)	28.0 [20.0–41.0]	30.0 [22.0–45.0]	0.210
Platelet counts (×10^9^/L)	234.0 [192.0–276.0]	245.0 [213.5–281.0]	0.011
Albumin (g/dL)	4.8 [4.6–4.9]	4.8 [4.6–5.0]	0.469
Fib-4 index	1.4 [1.0–1.9]	1.1 [0.8–1.5]	<0.001
Advanced fibrosis	27 (9.2)	10 (2.8)	0.001
Metabolic profile			
Fasting glucose (mg/dL)	105.0 [93.0–125.0]	101.0 [90.5–114.0]	0.001
HbA1c (%)	5.9 [5.5–6.6]	5.7 [5.4–6.1]	<0.001
Total cholesterol (mg/dL)	192.0 [160.0–216.0]	198.0 [169.0–226.5]	0.004
TG (mg/dL)	130.0 [98.0–182.0]	134.0 [95.0–191.5]	0.500
HDL-C (mg/dL)	49.0 [42.0–58.0]	50.0 [42.0–57.0]	1.000
LDL-C (mg/dL)	130.0 [97.0–153.0]	137.0 [106.5–163.5]	0.011
Statin therapy	55 (18.8)	56 (15.8)	0.002
Body composition profile			
Sarcopenia	42 (14.3)	44 (12.4)	0.543
Visceral obesity	185 (63.1)	159 (44.8)	<0.001
Myosteatosis	41 (14.0)	47 (13.2)	0.870
Cardiovascular profile			
ASCVD risk score	14.3 [9.3–22.8]	8.8 [4.0–14.9]	<0.001
CAC score	82.5 [24.6–220.0]		
CAC grade			
Mild (CAC score, 1–100)	165 (56.3)		
Moderate (CAC score, 101–300)	64 (21.8)		
Severe (CAC score, >300)	64 (21.8)		

ALT, alanine aminotransferase; ASCVD, atherosclerotic cardiovascular disease; AST, aspartate aminotransferase; BMI, body mass index; CAC, coronary artery calcification; Fib-4 index, fibrosis-4 index; HDL-C, high-density lipoprotein-cholesterol; LDL-C, low-density lipoprotein-cholesterol; MAFLD, metabolic dysfunction-associated fatty liver disease; TG, triglyceride.

**Table 2:** Univariate analysis for presence and severe coronary artery calcification in patients with metabolic dysfunction-associated steatotic liver disease.

	Presence of CAC			Severe CAC		
Variables	OR	95% CI	*p*-Value	OR	95% CI	*p*-Value
Age	1.09	1.07–1.11	<0.001	1.10	1.07–1.14	<0.001
Male	1.88	1.27–2.80	0.002	1.19	0.64–2.40	0.601
Current smoker	1.49	1.07–2.09	0.020	1.11	0.64–1.99	0.724
Type 2 diabetes	2.19	1.56–3.08	<0.001	2.07	1.22–3.49	0.007
Statin therapy	1.23	0.82–1.86	0.314	1.41	0.72–2.58	0.290
Advanced fibrosis	3.50	1.72–7.72	<0.001	4.45	2.01–9.32	<0.001
Sarcopenia	1.18	0.75–1.86	0.469	1.18	0.48–2.17	0.844
Visceral obesity	2.25	1.48–3.47	<0.001	5.15	1.87–21.34	0.006
Myosteatosis	1.07	0.68–1.67	0.780	1.73	0.87–3.25	0.101

CAC, coronary artery calcification; CI, confidence interval; OR, odds ratio.

**Table 3:** Adjusted odds ratio of visceral obesity for presence and severity of CAC in patients with MASLD.

	Presence of CAC		Severe CAC	
	aOR (95% CI)	*p*-Value	aOR (95% CI)	*p*-Value
Unadjusted	2.25 (1.48–3.47)	<0.001	5.15 (1.87–21.34)	0.006
Model 1	1.44 (0.90–2.31)	0.130	3.75 (1.32–15.77)	0.030
Model 2	1.38 (0.86–2.23)	0.180	3.54 (1.25–14.90)	0.039
Model 3	1.67 (1.08–2.64)	0.024	3.74 (1.31–15.79)	0.032

Model 1, adjusted for age and sex; model 2, adjusted model 1 + smoking, statin therapy, type 2 diabetes, and advanced fibrosis; model 3, adjusted for ASCVD risk score. ASCVD, atherosclerotic cardiovascular disease; aOR, adjusted odds ratio; CAC, coronary artery calcification; CI, confidence interval; MASLD, metabolic dysfunction-associated steatotic liver disease.

## Data Availability

The data used to support the findings of this study are available from the corresponding author upon request.

## Supplementary Materials

The following supporting information can be downloaded at: https://www.mdpi.com/article/10.3390/diagnostics14202305/s1, Table S1: Baseline characteristics according to the presence of severe coronary artery calcification.
