# Supplementary material for: Visceral Obesity and Its Association with Severe Coronary Artery Calcification in Patients with Metabolic Dysfunction-Associated Steatotic Liver Disease
Source: Diagnostics (Basel). 2024 Oct 17;14(20):2305. doi: 10.3390/diagnostics14202305 (PMC11506773; doi:10.3390/diagnostics14202305)
Supplement: Supplementary file 1 [file diagnostics-14-02305-s001.zip › diagnostics-3235257-supplementary.pdf]

Supplementary Table S1. Baseline characteristics according to the presence of severe coronary artery calcification

	CAC > 300<br>n = 64 (9.9%)	CAC ≤ 300<br>n = 584 (90.1%)	*p*-Value
Demographic profile			
Age (years)	64.5 [61.0–69.5]	57.0 [51.0–62.0]	<0.001
Male	52 (81.2)	458 (78.4)	0.716
BMI (kg/m^2)	22.7 [22.0–26.3]	23.1 [22.0–26.4]	0.703
Waist circumference (cm)	88.1 [83.8–95.5]	90.0 [85.2–95.8]	0.563
Type 2 diabetes	29 (45.3)	167 (28.6)	0.009
Hypertension	48 (75.0)	373 (63.9)	0.102
Current smoker	45 (70.3)	398(68.2)	0.833
Biochemical profile			
AST (U/L)	32.5 [23.5–45.0]	27.0 [21.0–36.0]	0.005
ALT (U/L)	29.0 [20.5–41.0]	29.0 [21.0–44.0]	0.853
Platelet counts (×10^9/L)	227.5 [188.5–275.0]	241.0 [205.0–279.5]	0.159
Albumin (g/dL)	4.7 [4.5–5.0]	4.8 [4.6–5.0]	0.136
Fib-4 index	1.7 [1.1–2.4]	1.2 [0.9–1.6]	<0.001
Advanced fibrosis	11 (17.2)	26 (4.5)	<0.001
Metabolic profile			
Fasting glucose (mg/dL)	107.0 [93.0–128.0]	102.0 [91.0–119.0]	0.100
HbA1c (%)	6.0 [5.5–6.7]	5.7 [5.4–6.3]	0.021
Total cholesterol (mg/dL)	178.5 [144.5–215.0]	196.5 [167.5–224.0]	0.021
TG (mg/dL)	121.5 [98.0–179.0]	134.0 [95.0–188.0]	0.403
HDL-C (mg/dL)	47.0 [41.0–59.5]	50.0 [42.0–57.0]	0.601
LDL-C (mg/dL)	120.0 [87.0–153.5]	134.0 [105.0–159.0]	0.036
Use of statin	14 (21.9)	97 (16.6)	0.023
Body composition profile			
Sarcopenia	9 (14.1)	77 (13.2)	0.998
Visceral obesity	46 (71.9)	298 (51.0)	0.002
Myosteatosis	13 (20.3)	75 (12.8)	0.143
Cardiovascular profile			
ASCVD risk score	19.4 [12.2–33.0]	10.8 [5.3–16.9]	<0.001
CAC score	687.8 [450.0–933.4]	0.0 [0.0–29.0]	<0.001

ASCVD, atherosclerotic cardiovascular disease; AST, aspartate aminotransferase; BMI, body mass index; CAC, coronary artery calcification; Fib-4 index, fibrosis-4 index; HDL-C, high-density lipoprotein-cholesterol; LDL-C, low-density lipoprotein-cholesterol; MAFLD, metabolic dysfunction-associated fatty liver disease; TG, triglyceride.
